# Rapid Degeneration of Noncoding DNA Regions Surrounding *SlAP3X/Y* After Recombination Suppression in the Dioecious Plant *Silene latifolia*

**DOI:** 10.1534/g3.113.008599

**Published:** 2013-10-11

**Authors:** Kotaro Ishii, Rie Nishiyama, Fukashi Shibata, Yusuke Kazama, Tomoko Abe, Shigeyuki Kawano

**Affiliations:** * RIKEN Nishina Center, Wako, Saitama 351-0198, Japan; † RIKEN Center for Sustainable Resource Science, Yokohama, Kanagawa 230-0045, Japan; ‡ Institute of Plant Science and Resources, Okayama University, Kurashiki 710-0046, Japan; § RIKEN Innovation Center, Wako, Saitama 351-0198, Japan; ** Department of Integrated Biosciences, Graduate School of Frontier Sciences, University of Tokyo, Kashiwa, Chiba 277-8562, Japan

**Keywords:** *SlAP3*, transposon, sequence divergence, sex chromosome, *Silene latifolia*

## Abstract

*Silene latifolia* is a dioecious plant with heteromorphic XY sex chromosomes. Previous studies of sex chromosome–linked genes have suggested a gradual divergence between the X-linked and the Y-linked genes in proportion to the distance from the pseudoautosomal region. However, such a comparison has yet to be made for the noncoding regions. To better characterize the nonrecombining region of the X and Y chromosomes, we sequenced bacterial artificial chromosome clones containing the sex chromosome–linked paralogs *SlAP3X* and *SlAP3Y*, including 115 kb and 73 kb of sequences, respectively, flanking these genes. The synonymous nucleotide divergence between *SlAP3X* and *SlAP3Y* indicated that recombination stopped approximately 3.4 million years ago. Sequence homology analysis revealed the presence of six long terminal repeat retrotransposon-like elements. Using the nucleotide divergence calculated between left and right long terminal repeat sequences, insertion dates were estimated to be 0.083–1.6 million years ago, implying that all elements detected were inserted after recombination stopped. A reciprocal sequence homology search facilitated the identification of four homologous noncoding DNA regions between the X and Y chromosomes, spanning 6.7% and 10.6% of the X chromosome–derived and Y chromosome–derived sequences, respectively, investigated. Genomic Southern blotting and fluorescence *in situ* hybridization showed that the noncoding DNA flanking *SlAP3X/Y* has homology to many regions throughout the genome, regardless of whether they were homologous between the X and Y chromosomes. This finding suggests that most noncoding DNA regions rapidly lose their counterparts because of the introduction of transposable elements and indels (insertion–deletions) after recombination has stopped.

Many plants, including the model plant *Arabidopsis thaliana*, are bisexual. However, approximately 6% of angiosperms are dioecious, meaning that male plants produce only male flowers and female plants produce only female flowers (Renner and Ricklefs 1995). Some dioecious plants have sex chromosomes with varying sizes and forms. *Carica papaya* (60 Mb) (Ming *et al.* 2008) and *Asparagus officinalis* (65 Mb) (Telgmann-Rauber *et al.* 2007), for example, have homomorphic sex chromosomes. In contrast, *Marchantia polymorpha* (X: 20 Mb; Y: 10 Mb) (Yamato *et al.* 2007) and *Rumex acetosa* (X: 740 Mb; Y_1_: 520 Mb; Y_2_: 410 Mb) (Mosiolek *et al.* 2005) have heteromorphic sex chromosomes. In *Silene latifolia*, sex is determined by heteromorphic sex chromosomes, with a pair of X chromosomes in females and both an X chromosome and a Y chromosome in males. The Y chromosome is estimated to be 570 Mb (Široký *et al.* 2001; Liu *et al.* 2004), representing approximately 9% of the total genome (Matsunaga *et al.* 1994), and is 1.4-times larger than the X chromosome (Matsunaga *et al.* 1994). Thus, the X and Y chromosomes of *S. latifolia* provide a good opportunity to study the evolutionary history of heteromorphic sex chromosomes during plant evolution.

Sex chromosomes have evolved independently in many plant groups (Charlesworth 2002). Sex chromosomes are thought to have originated from a pair of autosomes with two sexually antagonistic mutations in plants and in animals. It is also assumed that the chromosomal region harboring these mutations was subject to selection-driven suppression of recombination (Charlesworth 2013), which may have been facilitated by chromosomal inversions (Lemaitre *et al.* 2009; Wang *et al.* 2012) and translocations (Charlesworth and Charlesworth 1980). In mammals, such nonrecombining genomic regions are known to have expanded over evolutionary time (Iwase *et al.* 2003).

One way to estimate the age of sex chromosomes is to study X-Y divergence (Charlesworth 2013). Estimating the date of sex chromosome emergence is possible when using the sex-determining gene itself and the use of silent site divergence of an X-Y gene pair allows for estimation of the time when recombination stopped between the two chromosomal regions in which the gene pair resides. Using several X-Y gene pairs, dates associated with recombination suppression have been estimated in species of mammals (Lahn and Page 1999), birds (Lawson-Handley *et al.* 2004; Nam and Ellegren 2008), and plants [*S. latifolia* (Bergero *et al.* 2007) and *C. papaya* (Wang *et al.* 2012)], revealing differences in the estimated dates based on the chromosomal regions in which the pairs were located. Chromosomal regions exhibiting different silent site sequence divergence estimates are referred to as “evolutionary strata” (Lahn and Page 1999). The oldest stratum in *S. latifolia* is thought to have appeared 5–10 million years ago (MYA) (Nicolas *et al.* 2005). By mapping eight X-linked genes, it has been reported that silent site divergence between X-Y gene pairs increased in proportion to the distance of the gene pair from the pseudoautosomal region (PAR), and it has been suggested that recombination between the X and Y chromosomes stopped in progressive steps that formed two evolutionary strata (Bergero *et al.* 2007). Bergero *et al.* (2013) showed another stratum that was formed by additions of genome regions. However, previous studies focused on the comparison of only X-Y gene pairs or the accumulation of repetitive sequences at the chromosomal level (Kejnovsky *et al.* 2009). Thus, the nonrecombining regions that comprise each evolutionary stratum remain uncharacterized.

The *S. latifolia* MADS box gene, *SlAP3*, which exhibits significant similarity to *A. thaliana APETALA3*, was first isolated by Matsunaga *et al.* (2003) and was found to occur in two copies, *SlAP3Y* and *SlAP3A*. *SlAP3Y* has only been detected in male plants when genomic PCR is performed using *SlAP3Y*-specific primers and is thus known to be located on the Y chromosome. In contrast, *SlAP3A* was thought to be located on the autosome, because this gene was first detected by genomic PCR using *SlAP3A*-specific primers and flow-sorted X chromosomes and autosomes (Matsunaga *et al.* 2003). It was also later amplified by Cegan *et al.* (2010) by using microdissected X chromosomes, but not microdissected autosomes; the gene has since been renamed *SlAP3X*. Reaching the same conclusion, Nishiyama *et al.* (2010) conducted a segregation analysis of *SlAP3X* in which different lines of *S. latifolia* were crossed and the segregation of the male parent–derived sequence was examined. The initial misunderstanding of *SlAP3X* mainly results from the use of a localization test of flow-sorted chromosomes derived from cultured root cells in which the translocation including *SlAP3X* would have occurred. Cegan *et al.* (2010) compared promoter sequences of *SlAP3X* and *SlAP3Y* and revealed that a specific sequence was inserted in the *SlAP3Y* promoter. This inserted sequence shows some homology to the promoter sequence of *MROS1*, which is specifically expressed in male plants (Matsunaga *et al.* 1996, 1997). The divergence of noncoding regions is possibly a driving force behind the degeneration of X and Y chromosomes, resulting in differential expression of X-linked and Y-linked genes.

Based on the synonymous nucleotide divergence calculated in the coding regions of *SlAP3X* and *SlAP3Y*, we estimated that recombination between the two genes stopped approximately 3.4 MYA. Comparison of intron sequences within the pair of genes revealed that homologous regions have been segmentalized by insertions and deletions (indels) and contain repetitive sequences. Additionally, we compared sequences surrounding the gene pair; predictably, the transposons classes observed in these regions differed between the X and Y chromosomes. We estimated insertion times of predicted long terminal repeat (LTR) retroelements based on the nucleotide divergence between left and right LTRs, which implied that they had likely been inserted in these regions after recombination between *SlAP3X* and *SlAP3Y* stopped. These data provide the first evidence related to nucleotide resolution of sequence divergence between a pair of noncoding DNA regions on the sex chromosomes of *S. latifolia* after recombination stopped.

## Materials and Methods

### Plant materials

An inbred *S. latifolia* line, the K line (Kazama *et al.* 2003), was used for all experiments. A second line, the B line, was used for linkage mapping of the X chromosome. The original plants of K and B lines were provided by the University of Oslo Botanical Garden, Norway (K line), and the Royal Botanic Gardens, Kew, United Kingdom (B line). F_1_ progeny of the two phyletic lines were obtained by crossing a male plant of the K line with a female plant of the B line. F_2_ progeny were generated by crossing an F_1_ male with an F_1_ female. Plants were grown in pots in a regulated chamber at 23° under a 16:8-hr light/dark cycle. Young leaves were used for genomic DNA isolation.

### Bacterial artificial chromosome sequencing

The clone 2b5E (renamed as 13d11Eb) containing *SlAP3X* was isolated from a male *S. latifolia* bacterial artificial chromosome (BAC) library, as described by Ishii *et al.* (2010), using the 4D-PCR method (Asakawa *et al.* 1997; Ishii *et al.* 2008). All BAC clones (13d11Ea, 13d11Eb, and 7a8D) were purified using the PowerPrep HP Plasmid Purification Kit from OriGene Technologies (Rockville, MD) and were further purified by CsCl gradient ultracentrifugation. Emulsion PCR and 454 sequencing were performed on the Roche 454 FLX platform according to manufacturer’s instructions. Sequence assembly was performed using the GS *De novo* Assembler. A summary of the sequencing data is shown in Supporting Information, Table S1. Contigs consisting of more than 20 reads were first extracted and used to construct supercontigs in Sequencher (Gene Codes, Ann Arbor, MI). Contigs of 13d11Ea and 13d11Eb were analyzed together. All gaps between the contigs were PCR-amplified and sequenced by the Sanger sequencing method.

### Sequence analysis

Alignments of intron sequences of *SlAP3X* and *SlAP3Y* were conducted using the Needle program within the EMBOSS 6.5 package (Rice *et al.* 2000) with default settings, except that the gap penalty was set to 30.0 and the extend penalty was set to 0.0. Dot plot analyses were performed using HarrPlot 3.1.1 included in GENETYX version 11 with the unit size to compare set to 10 and the dot plot matching number set to 8. Homology searches were performed with the nucleotide sequence database of the National Center for Biotechnology Information using the TBLASTX program and with the repetitive sequence database, Repbase (Jurka *et al.* 2005) of the Genetic Information Research Institute (taxon: Viridiplantae), using the BLASTN program. These programs are contained in the BLAST+ package (Camacho *et al.* 2009). Repetitive sequences, including LTRs, simple repeats, and other repeat sequences, were detected by homology searches of the 13d11E or 7a8D sequences with their own sequences using the BLASTN program. The E-value threshold was set to 1E−10. Homologous regions were detected from homology searches of the 13d11E or 7a8D sequences using the BLASTN program. Thresholds for hit length, E-value, and identity were set to 500 bp, 1E−50, and 80%, respectively. The pair of coding regions of *SlAP3X* and *SlAP3Y*, the pairs of LTRs, and the pairs of homologous regions between the 13d11E and 7a8D sequences were aligned using the program MUSCLE (Edgar 2004). The synonymous nucleotide distance (k) between the coding regions of *SlAP3X* and *SlAP3Y* was estimated using the Kumar method (Nei and Kumar 2000). The nucleotide distances (k) of the pairs of the LTRs and the pairs of the homologous regions were estimated using the Tamura-Nei model (Tamura and Nei 1993) within MEGA5 (Tamura *et al.* 2011). An average substitution rate (r) of 1.8 × 10^−8^ substitutions per synonymous site per year (Du *et al.* 2010) was used for calculations. The time (T) since recombination stopped between *SlAP3X* and *SlAP3Y* was estimated using the following formula: T = k/2r.

### Genomic Southern blot analysis

Southern blot analysis methods were modified from those described previously (Kazama *et al.* 2006). Genomic DNA was extracted from *S. latifolia* leaves using a Nucleon PhytoPure Genomic DNA Extraction Kit (GE Healthcare, Little Chalfont, England). Genomic DNA (15 μg) was digested with *Eco*RI for 12 hr. The concentrations of the digests were measured, and the digests were loaded onto a 1.0% (w/v) agarose gel and then transferred to an Immobilon-Ny+ membrane (Merck, Darmstadt, Germany). Hybridization and signal detection were performed using the AlkPhos Direct Labeling and Detection System (GE Healthcare), and each probe was amplified individually by PCR with locus-specific primers (Table S2). The hybridized membranes were visualized using CDP-*Star* (GE Healthcare) at room temperature with appropriate exposure times.

### FISH analysis

Fluorescent *in situ* hybridization (FISH) was performed as previously described (Ishii *et al.* 2010), with minor modifications. Two probes for the homologous (I) and nonhomologous (a) regions were prepared from the same PCR products produced in the genomic Southern blot analysis using DIG-Nick and the Biotin-Nick Translation Mix (Roche Diagnostics, Basel, Switzerland), respectively. Chromosomal DNA was denatured at 70° for 1 min in 2× SSC buffer containing 70% formamide. The chromosome preparations were dehydrated immediately by 5-min treatments with 70% ethanol at −20° and 100% ethanol at room temperature. The preparations were then dried for 30 min at room temperature. Each slide was loaded with 10 µL of the hybridization mixture containing 12.5 ng probe, 50% formamide, 10% dextran sulfate, and 2× SSC buffer. The slides were washed twice in 2× SSC buffer at 37°. The signals were fluoresceinated with antidigoxigenin-rhodamine (Roche Diagnostics) and avidin-Alexa Fluor 488 (Molecular Probes, Eugene, Oregon) for the digoxigenin-labeled probe and biotin-labeled probe, respectively. Preparations were counterstained with 4′,6-diamidino-2-phenylindole (DAPI). The fluorescent Alexa Fluor 488, DAPI, and rhodamine images were detected consecutively under a Leica Q550 cytogenetic workstation (Leica Microsystems, Wetzlar, Germany) equipped with a black-and-white charged-coupled device camera (CoolSNAP HQ; Nippon Roper, Tokyo, Japan) using the SpectraVision filters SpectrumGreen, SpectrumDAPI, and SpectrumOrange, respectively (Vysis, Chicago, IL).

### X chromosome linkage mapping

To genotype the population, genomic PCRs were performed using K line–specific primers for *SlAP3X*, *SlX1*, and *SlX4* (Table S3) on 96 F_2_ progeny derived from a cross between a K line male and a B line female. Restriction fragment length polymorphism analysis was used to genotype the *DD44X* locus. Genomic DNA fragments were amplified with *DD44X*-specific primers (Table S3) from the 96 F_2_ progeny, digested with *Nco*I, and analyzed by electrophoresis. The Kosambi mapping function was used to compute genetic distances in cM (Kosambi 1944).

## Results

### Structure of the *SlAP3X/Y* transcriptional region

The number of synonymous nucleotide divergences per synonymous sites between translated regions of *SlAP3X* and *SlAP3Y* was 0.12 (SE, 0.034). Nicolas *et al.* (2005) estimated that in the oldest evolutionary strata, recombination stopped 5–10 MYA by using the average synonymous substitution rates of two genes (1.8 × 10^−8^ substitutions per synonymous site per year) in the Brassicaceae species (Koch *et al.* 2000). Similarly, we estimated that recombination between *SlAP3X* and *SlAP3Y* stopped 3.4 MYA (SE, 0.94).

*SlAP3X* has five introns and *SlAP3Y* has six introns (Nishiyama *et al.* 2010). Exon V and exon VI of *SlAP3Y* are homologous to the anterior half and the posterior half of exon V of *SlAP3X* (Nishiyama *et al.* 2010), respectively, which indicated that intron 5 of *SlAP3Y* was inserted after recombination stopped. Thus, the five pairs of introns shown in Table 1 were considered to be homologous because they underwent recombination before recombination suppression. Nishiyama *et al.* (2010) previously reported that intron 2 of *SlAP3X* was 561 bp long, whereas intron 2 of *SlAP3Y* was 24.4 kb long, containing two retroelements and one telomere-like sequence. Therefore, we concluded that the majority of intron 2 of *SlAP3Y* was inserted after recombination stopped. We aligned each pair of introns and calculated the similarities. Although lengths of the paired introns differed between *SlAP3X* and *SlAP3Y*, calculated sequence similarities (excluding gaps) were between 76% and 82% (Table 1).

**Table 1:** Homology between introns of *SlAP3X* and *SlAP3Y*

*SlAP3X* (13d11E)				*SlAP3Y* (7a8D)				
Intron	Start, bp	End, bp	Length, bp	Intron	Start, bp	End, bp	Length, bp	Identity (%)
1	86,023	86,127	105	1	34,286	34,402	117	78
2	86,195	86,755	561	2	34,470	58,893	24,424	78
3	86,818	86,912	95	3	58,956	59,297	342	76
4	87,013	87,242	230	4	59,400	59,784	385	76
				5	59,827	59,937	111	—
5	87,330	87,430	101	6	59,983	60,375	393	82

**Figure 1:**
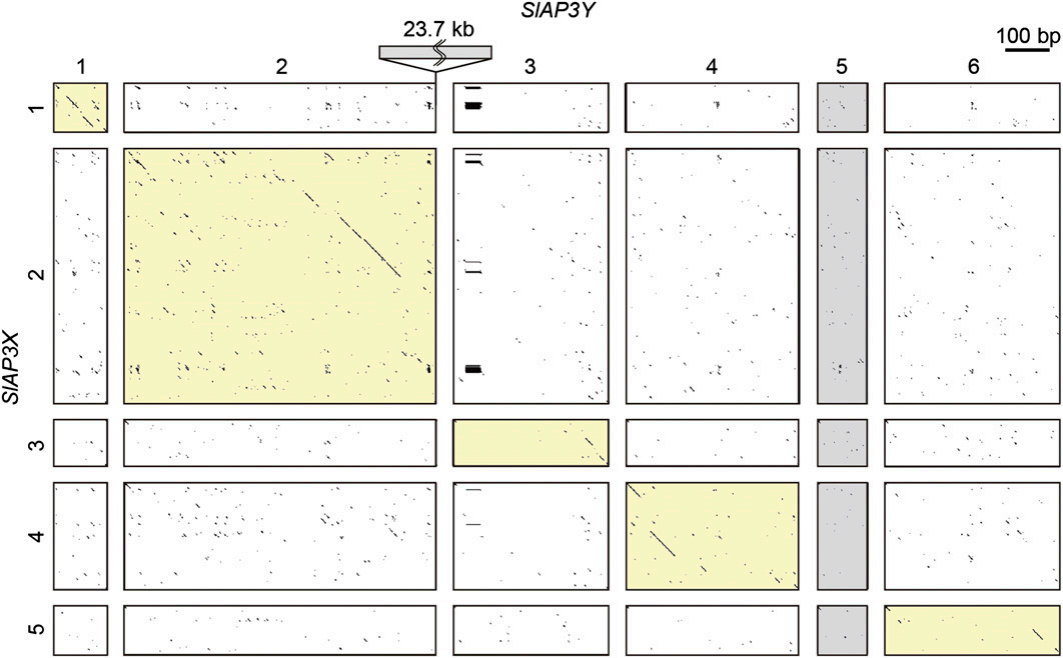
Dot plot analysis of introns of *SlAP3X* and *SlAP3Y*. Each intron sequence of *SlAP3X* (vertical) is compared to that of *SlAP3Y* (horizontal). The first 700 bp of *SlAP3Y* intron 2 is shown. Numbers above each axis indicate the ordinal number of each intron. Yellow squares indicate comparisons for which homologous regions were detected.

**Figure 2:**
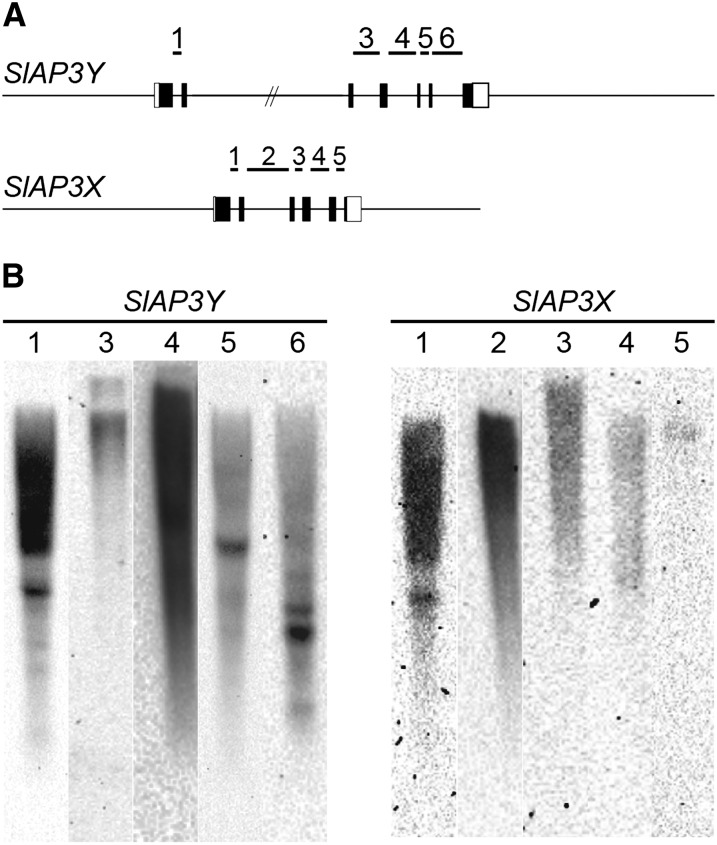
Genomic Southern blot analysis of *SlAP3X* and *SlAP3Y*. (A) Schematics of genomic sequences of *SlAP3X* and *SlAP3Y*. White and black boxes indicate untranslated regions and exons, respectively. Numbers indicate intron numbers of the genes and bold lines indicate probe sequence coverage of each intron for Southern hybridization. (B) Genomic Southern blot analysis of introns of *SlAP3X* and *SlAP3Y*. Numbers above lanes indicate intron numbers of the genes corresponding to those in (A).

We conducted dot plot analyses of each pair of introns (Figure 1). All five pairs contained regions of sequence homology, which implied that the pairs were homologous before recombination suppression. However, these homologous regions did not span the full lengths of the introns but were interspersed like island chains. To investigate whether introns consisted of unique or repetitive sequences, we designed probes inside the introns and performed genomic Southern blot analyses (Figure 2). The probes designed for the introns showed three patterns: a strong smear pattern (introns 1 and 2 of *SlAP3X* and introns 1 and 4 of *SlAP3Y*); a weak smear pattern (intron 3, 4, and 5 of *SlAP3X* and introns 3 of *SlAP3Y*); and a multiple-banded pattern (introns 5 and 6 of *SlAP3Y*). From this, it was revealed that all introns contained repetitive sequences; the distinctness of these patterns implied that each intron contained a different type of repeat sequence.

### Transposons in the peripheral regions of *SlAP3X/Y*

**Figure 3:**
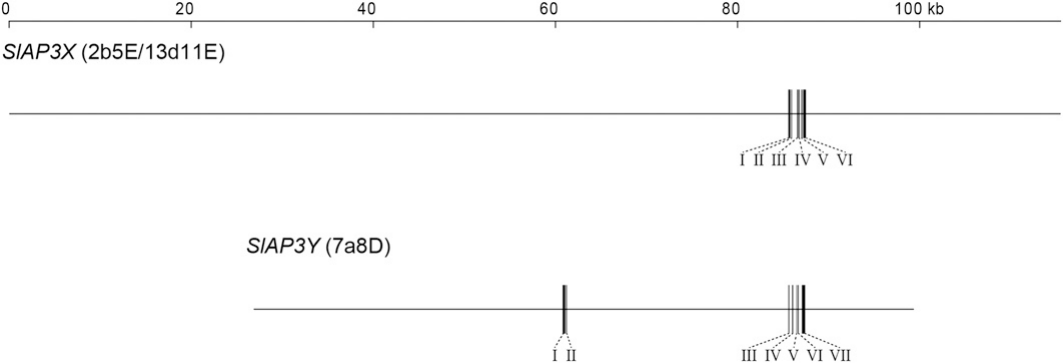
Schematics of BAC sequences. Horizontal lines indicate entire sequences of each BAC clone drawn to scale as indicated. Black bars indicate exons, which are labeled with Roman numerals.

To obtain sequences in the peripheral regions of *SlAP3X* and *SlAP3Y*, we sequenced the following three BAC clones using the 454-FLX sequencing platform: 13d11Ea containing *SlAP3X*; 7a8D containing *SlAP3Y* (Nishiyama *et al.* 2010); and 2b5E containing *SlAP3X* (isolated in this study). The sequences of 13d11Ea and 2b5E spanned the same chromosomal region, which indicated that they were derived from the same genome fragment; 2b5E was thus renamed 13d11Eb. After the vector sequence was removed, we obtained a 115,665-bp sequence flanking *SlAP3X* (13d11E) and a 72,683-bp sequence flanking *SlAP3Y* (7a8D) (Figure 3).

We conducted TBLASTX searches of the National Center for Biotechnology Information nucleotide database using the 13d11E and 7a8D sequences as the query sequences (Table S4 and Table S5). In 13d11E, 11 regions were homologous to the coding regions of LTR retrotransposons and one region was homologous to a coding region of a DNA transposon. In 7a8D, six regions were homologous to coding regions of LTR retrotransposons and two regions were homologous to LINEs. BLASTN searches of the Genetic Information Research Institute Repbase database (taxon: Viridiplantae) using 13d11E and 7a8D as query sequences revealed that 22 regions in 13d11E were homologous to LTR retrotransposons, and seven regions in 7a8D were homologous to LTR retrotransposons (Table S6). The regions determined to be homologous to transposons by BLASTN were also identified using TBLASTX. Both 13d11E and 7a8D sequences had regions homologous to gypsy retrotransposons and copia retrotransposons. Regions homologous to LINEs were found only in the 7a8D sequence. Genes other than transposons and *SlAP3X/Y* were not detected in either 13d11E or 7a8D.

**Figure 4:**
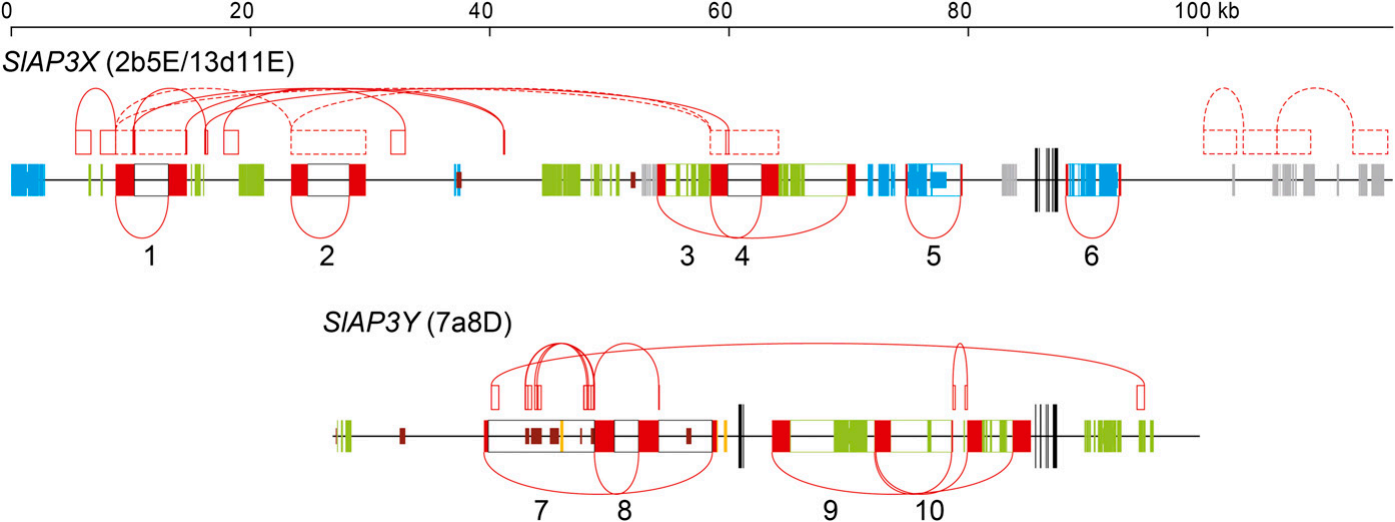
Transposable elements in the BAC sequences. Entire sequences of the BAC clones and the locations of exons are displayed in the same manner as in Figure 3. Blue, green, gray, and orange boxes on the horizontal line indicate regions homologous to copia-like retrotransposons, gypsy-like retrotransposons, DNA transposons, and LINEs, respectively. Bold and thin boxes are detected by the TBLASTX and BLASTN searches, respectively. White boxes sandwiched by pairs of red boxes connected by arcs on the horizontal line indicate the locations of LTR retrotransposon-like elements. The numbers below LTRs correspond to those listed in Table 2. Brown boxes indicate simple repeat sequences. Pairs of boxes outlined in solid red lines and connected by an arc above the horizontal line indicate other repetitive sequences. Pairs of boxes outlined with dashed red lines indicate homologous regions in which evolutionarily related elements have been inserted.

To identify LTR sequences sandwiching regions homologous to LTR retrotransposons, we performed pairwise BLASTN searches of 13d11E and 7a8D within BACs (Figure 4). Six and four pairs of sequences, homologous in the forward direction, were found in 13d11E and 7a8D, respectively, and were predicted to be LTR sequences (Table 2 and Figure 4). All LTR retrotransposon–like elements did not contain any intact genes and were presumed to be nonautonomous. At one and two loci in 13d11E and 7a8D, respectively, two LTR retrotransposon–like elements were identified with nested structures (Figure 4). The BLASTN searches also revealed the existence of two simple repeat sequences in 13d11E and seven simple repeat sequences in 7a8D. Six out of seven simple repeat sequences in 7a8D were found in one LTR retrotransposon–like element. The majority of sequences of the three LTR retrotransposon–like elements in 13d11E were homologous to one another; similar results were observed for two DNA transposon–like elements in 13d11E, which indicated that they were genetically related to each other.

**Table 2:** Predicted LTR retroelements and estimated insertion times

							BLASTN			
Clone	ID	Type	Start, bp	End, bp	Length, bp	Left LTR Length, bp	E-value	Identity, %	K (SE)	Estimated Insertion Time, MYA (SE)
13d11E (*SlAP3X*)	1	Unknown	8721	14,676	5956	1548	0	99	0.0065 (0.0021)	0.18 (0.057)
	2	Unknown	23,393	29,638	6246	1387	0	99	0.011 (0.0028)	0.30 (0.079)
	3	Gypsy	54,004	70,569	10,886	715	0	99	0.0080 (0.0057)	0.22 (0.16)
	4	Unknown	58,455	64,134	5680	1450	0	96	0.027 (0.0044)	0.74 (0.12)
	5	Copia	74,749	79,514	4766	169	7E−65	93	0.045 (0.017)	1.2 (0.48)
	6	Copia	88,141	92,782	4642	208	5E−86	95	0.057 (0.018)	1.6 (0.50)
7a8D (*SlAP3Y*)	7	Unknown	12,651	32,108	14,093	366	4E−153	94	0.044 (0.012)	1.2 (0.32)
	8	Unknown	21,888	27,252	5365	1670	0	99	0.0030 (0.0013)	0.083 (0.037)
	9	Gypsy	36,730	58,367	12,663	1538	0	97	0.022 (0.0039)	0.62 (0.11)
	10	Gypsy	45,305	54,279	7770	1345	0	97	0.024 (0.0043)	0.65 (0.12)

K, nucleotide divergence between left and right LTRs; BLASTN, homology between the left and right LTR sequences calculated by the BLASTN program.

### Transposons inserted after recombination suppression

**Figure 5:**
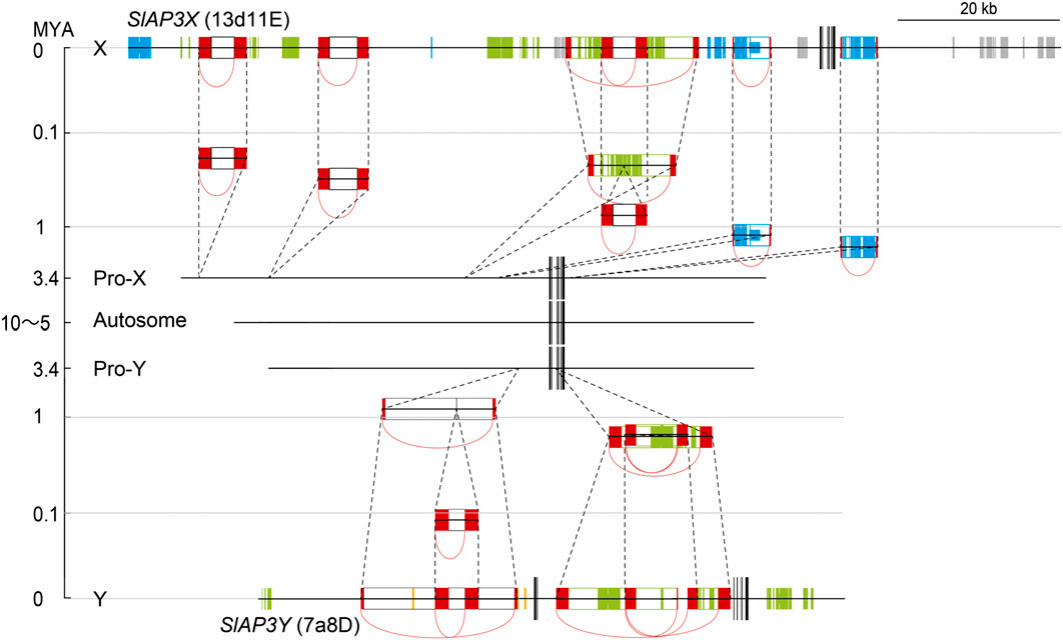
Estimated insertion dates of LTR retroelements. The horizontal line in the middle of the vertical axis indicates the constructed genomic sequence peripheral to the ancient *SlAP3X/Y* before recombination suppression. The upper half and lower half of the figure show temporal changes of genomic sequence peripheral to *SlAP3X* and *SlAP3Y*, respectively, by insertion of LTR retroelements in single logarithmic plots. LTR retroelements are displayed in the same manner as in Figure 4. Horizontal lines drawn through the center of boxes indicate the estimated insertion date of LTR retroelements. Dashed lines indicate insertion sites.

Du *et al.* (2010) estimated the insertion dates of LTR retrotransposons using genetic distances calculated between left and right LTR sequences. In the same manner, we calculated the genetic distances between the left and right LTR sequences of the LTR retrotransposon–like elements detected in 13d11E (*SlAP3X*) and 7a8D (*SlAP3Y*), from which we estimated the dates when they were inserted (Table 2 and Figure 5). All the investigated LTR retrotransposon–like elements were estimated to be inserted after recombination between *SlAP3X* and *SlAP3Y* had stopped. Even the oldest one, which is the copia retrotransposon-like element, was presumed to be inserted 1.6 MYA (SE, 0.50). The dates when the LTR retrotransposon–like elements were inserted differed depending on the kind of element. The two copia retrotransposon-like elements in the 13d11E sequence were assumed to be inserted 1.2 MYA (SE, 0.48) and 1.6 MYA (SE, 0.50). The one and two gypsy retrotransposon-like elements in the 13d11E and 7a8D sequences were thought to be inserted 0.22 MYA (SE, 0.16), 0.62 MYA (SE, 0.11), and 0.65 MYA (SE 0.12), respectively. The three unknown retrotransposon-like elements in 13d11E were considered to be inserted 0.18 MYA (SE, 0.057), 0.30 MYA (SE, 0.079), and 0.74 MYA (SE 0.12), respectively. One and two pairs of nested LTR retrotransposon–like elements were detected in 13d11E and 7a8D, respectively (Figure 5). It was natural to assume that the inner element was inserted after the outer element. With regard to the two pairs in the 7a8D sequence, the estimated insertion dates of the inner elements were more recent than those of the outer elements. The estimated insertion time of the inner element of the pair identified in 13d11E was older than that of the outer element, but the two insertion dates were determined to be close and not significantly different.

### Homology between noncoding DNA regions of the X and Y chromosomes

**Figure 6:**
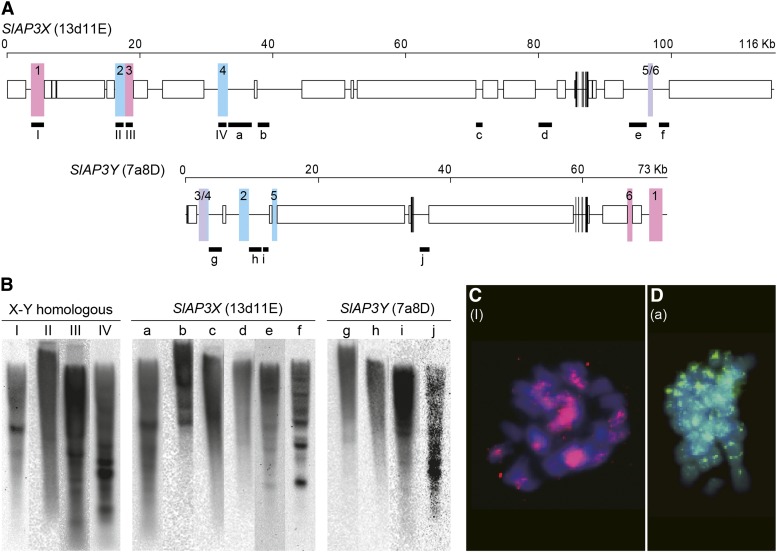
Noncoding DNA regions in peripheral regions of *SlAP3X* and *SlAP3Y*. (A) Maps of noncoding DNA regions. Entire sequences of the BAC clones and the locations of exons are displayed in the same manner as in Figure 3. White boxes indicate coding regions of transposable elements or regions sandwiched by a pair of LTRs. Pink and blue boxes indicate noncoding and homologous regions between 13d11E and 7a8D in the forward direction and reverse direction when *SlAP3X* and *SlAP3Y* are aligned in the same direction, respectively. Numbers on the boxes indicate correspondence between homologous regions. Horizontal lines indicate regions that are noncoding but not homologous between 13d11E and 7a8D. Black bold lines indicate probe regions for genomic Southern hybridization and FISH. Roman numbers and lower-case characters below bold lines correspond to those in (B), (C), and (D). (B) Genomic Southern blot analysis of noncoding DNA regions. The membranes used in Figure 2B were hybridized with each PCR product of noncoding DNA regions. Roman and Arabic numbers above lanes indicate probe regions corresponding to those in (A). (C) FISH result for the probe of the region (I) that covered the noncoding and X-Y homologous sequence. (D) FISH result for the probe of the region (a) spanning the noncoding and nonhomologous sequence.

The noncoding DNA regions around *SlAP3X* and *SlAP3Y*—except for the transposons that were inserted after recombination stopped—should have been homologous. We attempted to reveal structural changes of the sex chromosomes after recombination suppression by investigating how much homologous sequence remained. To detect homologous regions between the X and Y chromosomes, we conducted BLASTN searches of 13d11E (*SlAP3X*) using 7a8D (*SlAP3Y*) as the query sequence. Homologous regions were scattered around *SlAP3X/Y* (Table 3 and Figure 6A). The total length of the homologous regions in the 13d11E and 7a8D sequences were 7.7 kb (6.7%) and 7.7 kb (10.6%), respectively. We calculated the nucleotide divergence of each homologous sequence pair (Table 3). The divergences showed some variation, ranging between 0.085 (SE, 0.0085) (Table 3, ID 4) and 0.25 (SE, 0.029) (Table 3, ID 5), whereas that observed for the coding regions of *SlAP3X/Y* was 0.12 (SE, 0.034).

**Table 3:** Homologous regions between peripheral regions of *SlAP3X* and *SlAP3Y*

	13d11E (*SlAP3X*)			7a8D (*SlAP3Y*)						
ID	Start, bp	End, bp	Length, bp	Start, bp	End, bp	Length, bp	Direction	E-value	Identity, %	K (SE)
1	3586	5587	2002	71,985	70,005	1981	Inverted	0	73	0.14 (0.012)
2	16,253	17,744	1492	8055	9530	1474	Direct	0	76	0.11 (0.010)
3	17,735	18,992	1258	3248	1996	1253	Inverted	0	81	0.14 (0.014)
4	31,730	33,237	1508	1996	3467	1470	Direct	0	78	0.085 (0.0085)
5	96,491	97,215	725	67,473	66,715	759	Inverted	5.00E−166	78	0.25 (0.029)
6	96,492	97,219	728	13,016	13,789	772	Direct	4.00E−161	77	0.092 (0.013)

K, nucleotide divergence between the regions in 13d11E and 7a8D.

To investigate whether the homologous regions between 13d11E and 7a8D in the noncoding DNA regions were unique in the genome, we performed genomic Southern hybridizations with probes made from each PCR product of the homologous region in the 13d11E sequence (Table 3 and Figure 6B). Although probe sequences do not include transposons, all the hybridizations produced smeary patterns, which implied that these sequences were abundant in the genome. FISH performed using one of those sequences (Figure 6B, I) as a probe also yielded many signals on multiple chromosomes (Figure 6C). To investigate the uniqueness of noncoding DNA regions in the genome, which did not show homology to their counterparts on the opposite chromosome (Figure 6A, horizontal lines), we also analyzed genomic Southern hybridization data using PCR products generated from these DNA regions as probes (Figure 6B, a–j). All hybridizations yielded smeared patterns, which indicated that the genome included a large number of these sequences. FISH performed using one of these sequences (Figure 6B, d) as a probe also showed signals on multiple chromosomes, which supported the repetitive nature of the sequence (Figure 6D).

## Discussion

### Beginning of divergence between sequences surrounding *SlAP3X* and *SlAP3Y*

We estimated that recombination between *SlAP3X* and *SlAP3Y* stopped 3.4 MYA. In addition, we determined the dates of the subsequent insertions of transposons in this region. The molecular clock used for age estimations, which was the same as the one Nicolas *et al.* (2005) used, was based on the Brassicaceae species, which is currently the most reliable clock for *S. latifolia*. More accurate estimation will be possible when a molecular clock of more closely related species becomes available.

To confirm the location of *SlAP3X* within the nonrecombining region, we constructed a linkage map of the *S. latifolia* X chromosome based on the estimation of genetic distances (Table S7). The most probable order of the four genes from the PAR on the X chromosome was *SlX1*, *SlAP3X*, *DD44X*, and *SlX4*. The relative locations of genes on the X chromosome, integrated with the data presented by Bergero *et al.* (2013), are shown in Figure S1. Twenty percent divergence is typical for stratum 1 genes and 10% divergence is typical for stratum 2 genes (Cegan *et al.* 2010). *SlAP3X* was mapped between *DD44X* and *SlX1*, and the divergence between *SlAP3X* and Y was 12%, which implies that *SlAP3X* is located in stratum 2.

### Differentiation of sequences in sex chromosomes

Many diverged transposons were found in 13d11E (*SlAP3X*) and in 7a8D (*SlAP3Y*) (Figure 4); however, the sequence contents of these elements were determined to be different. For example, no significant similarity was found between retroelements identified in 13d11E (*SlAP3X*) and 7a8D (*SlAP3Y*), indicating either that these retroelements were independently inserted into 13d11E (*SlAP3X*) or 7a8D (*SlAP3Y*) after recombination stopped or that they had existed on the proto-sex chromosomes and were translocated to a distant region on only one of the chromosomes. Because the estimated insertion time of the oldest LTR retroelement (1.6 MYA) is more recent than the predicted time when recombination between *SlAP3X* and *SlAP3Y* (3.4 MYA) stopped, the former scenario is more likely to have occurred. Gschwend *et al.* (2012) compared the sequence of the *C. papaya* X chromosome with the autosomal counterpart of the related species, *Vasconcellea monoica*, and reported that sequences not only on the Y chromosome but also on the X chromosome expanded after recombination suppression. Obtaining the sequence of the *SlAP3* ortholog and its flanking region in *Silene vulgaris*, which is the closest related hermaphroditic species to *S. latifolia*, would enable us to better discern changes in the DNA sequence that occurred independently in the X and Y chromosomes.

All three unknown retrotransposon-like elements (ID 1, ID 2, and ID 4 in Table 2 and Figure 4) in 13d11E (*SlAP3X*) showed evidence of homology. In addition, the sequences of the two DNA transposon-like elements, which were nested in 13d11E (*SlAP3X*), were also homologous to one another (Figure 4). Thus, each group of transposons was concluded to have the same origin. The estimated insertion times of the three unknown retrotransposon-like elements in 13d11E (*SlAP3X*) were similar (0.18–0.74 MYA) (Table 2), suggesting that transposons of this type were translocated within a limited period. The estimated insertion times of the two copia retrotransposon-like elements in 13d11E (*SlAP3X*) were also similar (1.2 and 1.6 MYA) (Table 2). It is possible that each group of transposons has a shared origin but has lost homology over time. LINEs were detected only in the 7a8D sequence (Figure 4); however, according to the mapping of transposons by FISH analyses (Kejnovsky *et al.* 2009), they were distributed across the full length of both the X and Y chromosomes. It is possible that LINEs could be identified in the adjacent sequence of 13d11E (*SlAP3X*).

Yu *et al.* (2008) compared two pairs of BAC sequences derived from sex chromosomes of *C. papaya*. One pair of sequences (X BAC 61H02 and Y^h^ BAC 95B12) was thought to have stopped recombining 7.0 MYA, in that 81.6% and 74.5% of sequences of the X and Y^h^ chromosome had homology, respectively, and the identity between homologous sequences was 87.4%. The other pair of sequences (X BAC 53E18 and Y^h^ BAC 85B24) was estimated to have ceased recombination 1.9 MYA. In this example, 41.5% and 30.7% of sequences of the X and Y^h^ chromosome had homology, respectively, and the identity between homologous sequences was 83.6%. In the pair of sequences (13d11E and 7a8D) analyzed here for *S. latifolia*, which were assumed to have stopped recombining 3.4 MYA, 6.7% and 10.6% of sequences of the X and Y chromosomes had homology, respectively, and the average identity between homologous sequences was 76.9%. Differences in the percentages of the noncoding homologous region are thought to be, in part, attributable to differences in the lengths of the analyzed sequences. The lower percentages despite the identity may reflect a high rate of accumulation of transposable elements, which may have partially caused the heteromorphism of sex chromosomes in this species.

Regarding indels, Sundström *et al.* (2003) conducted comparative analysis on intron sequences of paralogs in primates and birds and revealed that in birds with female heterogamety, indels occurred in the Z chromosome approximately twice as often as in the W chromosome, and that in primates with male heterogamety, indels occurred at similar rates in both the X and Y chromosome. This indicated that both meiotic recombination and the number of cell divisions in reproductive cells play an important role in the occurrence of indels. It raises an interesting question regarding whether this theory is also applicable to plants in which reproductive cells are generated from vegetative cells, thus weakening the influence of the difference in the number of cell divisions between male and female reproductive cells. However, regarding transposable elements, a comparison between the 40-kb neo-X and 45-kb neo-Y sequences of *Drosophila miranda*, which is assumed to have emerged approximately 1 MYA, revealed that insertions of seven transposable elements occurred only in the neo-Y chromosome (Bachtrog 2005). It is assumed that transposable elements tend to accumulate more in the recombination suppression region of the Y chromosome. In the regions in *S. latifolia* analyzed in this study, transposable elements have highly accumulated not only in the Y chromosome but also in the X chromosome for 3.4 million years, preventing us from revealing whether the insertion of the transposable element was Y chromosome–biased. Obtaining continuous sequences between *SlAP3* and its neighboring genes in both X and Y chromosomes will enable us to find the answers to our questions.

### Origin of the noncoding DNA region

All sequences of noncoding DNA regions investigated in this study contained repetitive elements, irrespective of whether they were homologous between sex chromosomes (Figure 2B and Figure 6, B–D). Five noncoding DNA regions exhibited homology between the X and Y chromosomes (Table 3, IDs 1–4 and ID 6) and showed no significant differences in divergence from the synonymous nucleotide divergences observed for the translated regions of *SlAP3X* and *SlAP3Y*, as determined by the chi-square test. Because the divergence estimates for these regions along with *SlAP3X/Y* are consistent with their chromosomal location in stratum 2, it is considered that these regions are derived from ancestral chromosomes. However, one noncoding DNA region (Table 3, ID 5) had higher divergences (*P* = 0.0001 by chi-square test). Although the cause of the high divergence is uncertain, it is presumed that the sequences of the region have paralogous origins that had been duplicated in the past. Alternatively, as suggested in primates (Iwase *et al.* 2010), it could be possible that gene conversion occurred locally between the *SlAP3X* and *SlAP3Y* coding regions after recombination suppression, which would make divergence lower. With regard to noncoding DNA regions that were not homologous between the sex chromosomes, it was assumed that these sequences were inherited from ancestral chromosomes and that their counterparts in either the X chromosome or the Y chromosome were lost during deletion events.

### Evolutionary strata on the X chromosome in *S. latifolia*

Bergero *et al.* (2013) revealed that the X chromosome of *S. latifolia* have three evolutionary strata. However, because whole sequences of sex chromosomes have not been generated, it is uncertain whether the evolutionary strata on the X chromosome of *S. latifolia* have clear boundaries. Lemaitre *et al.* (2009) proposed that evolutionary strata on the human X chromosome were formed by inversions on the Y chromosome, which has been explained by the isochromatid model (Ranz *et al.* 2007), because boundary sequences between strata 4 and strata 5 and between PAR and strata 5 on the X chromosome are duplicated on the Y chromosome. According to the isochromatid model, two pairs of staggered single-strand breaks yield long 5′ overhangs, which are filled-in by DNA synthesis. When such breaks are repaired by nonhomologous end-joining, they can result in inversions flanked by inverted duplications of the sequences between the paired single-strand breaks (Ranz *et al.* 2007).

In two instances, we identified noncoding DNA regions that were present at two locations on one sex chromosome and only one homologous location on the second sex chromosome (Figure 6A). For example, region 3/4 in 7a8D (*SlAP3Y*) was homologous to region 3 and region 4 in 13d11E (*SlAP3X*). Region 2 in 7a8D, which was adjacent to region 3/4, was also homologous to region 2 in 13d11E, which was adjacent to region 3 (Figure 6A). These homologous noncoding DNA regions could be vestiges of inversions; however, definitive boundaries of sequence similarity that could be used to indicate evolutionary strata were not found.

In this study, we analyzed 116 kb and 73 kb of sequence from the X and Y chromosomes of *S. latifolia* and found that only 6.7% of the X chromosome–derived sequence and 10.6% of the Y chromosome–derived sequence were homologous between the two chromosomes. It was shown that sex chromosomes of *S. latifolia* were heteromorphic not only at the chromosomal level but also at the sequence level. We detected two locations in which homologous sequences between the X and Y chromosomes were duplicated, implying the occurrence of inversions. A wider comparison of noncoding DNA regions between the X and Y chromosomes, especially those near the PAR, will help delineate the boundaries of evolutionary strata and define differences in the degree of heteromorphy between these strata, which will be essential for gaining a broader understanding of the evolution of plant sex chromosomes.

## Supplementary Material

Supporting Information
