# Supplementary material for: Rapid Degeneration of Noncoding DNA Regions Surrounding SlAP3X/Y After Recombination Suppression in the Dioecious Plant Silene latifolia
Source: G3 (Bethesda). 2013 Oct 11;3(12):2121–30. doi: 10.1534/g3.113.008599 (PMC3852375; doi:10.1534/g3.113.008599)
Supplement: Supporting Information [file supp_3_12_2121__index.html]

Rapid Degeneration of Noncoding DNA Regions Surrounding SlAP3X/Y After Recombination Suppression in the Dioecious Plant Silene latifolia — Supporting Information 

# Rapid Degeneration of Noncoding DNA Regions Surrounding *SlAP3X/Y* After Recombination Suppression in the Dioecious Plant *Silene latifolia*

## Supporting Information for Ishii *et al.*, 2013

**Files in this Data Supplement:**

- Supporting Information - Figure S1 and Tables S1-S7 (PDF, 762 KB)
- Figure S1 - Genetic mapping of X-linked genes. (PDF, 331 KB)
- Table S1 - Summary of BAC sequencing. (PDF, 230 KB)
- Table S2 - Primer sets used for Southern blot analysis of introns. (PDF, 296 KB)
- Table S3 - Primer sets used for X chromosome linkage mapping. (PDF, 294 KB)
- Table S4 - Result of TBLASTX search using 13d11E as the query sequence. (PDF, 279 KB)
- Table S5 - Result of TBLASTX search using 7a8D as the query sequence. (PDF, 243 KB)
- Table S6 - Result of BLASTN searches. (PDF, 249 KB)
- Table S7 - Number and proportion (in parentheses) of recombinants (above the diagonal) and genetic distance (in cM, below the diagonal) between the four X-linked genes. (PDF, 242 KB)
