# Supplementary material for: Rapid Degeneration of Noncoding DNA Regions Surrounding SlAP3X/Y After Recombination Suppression in the Dioecious Plant Silene latifolia
Source: G3 (Bethesda). 2013 Oct 11;3(12):2121–30. doi: 10.1534/g3.113.008599 (PMC3852375; doi:10.1534/g3.113.008599)
Supplement: Supporting Information [file supp_g3.113.008599_008599SI.pdf]

G3: Genes | Genomes | Genetics

Rapid degeneration of noncoding DNA regions surrounding *SlAP3X/Y* after recombination suppression in a dioecious plant, *Silene latifolia*

Kotaro Ishii[*], Rie Nishiyama[§], Fukashi Shibata[†], Yusuke Kazama[‡], Tomoko Abe[*, ‡], and Shigeyuki Kawano[**]

[*]RIKEN Nishina Center, Wako, Saitama 351-0198, Japan

[§]RIKEN Plant Science Center, Yokohama, Kanagawa 230-0045, Japan

[†]Institute of Plant Science and Resources, Okayama University, Kurashiki 710-0046, Japan.

[‡]RIKEN Innovation Center, Wako, Saitama 351-0198, Japan

[**]Department of Integrated Biosciences, Graduate School of Frontier Sciences, The University of Tokyo, Kashiwa, Chiba 277-8562, Japan

Sequence data from this article have been deposited in the DNA Data Bank of Japan

(DDBJ) under accession nos. AB771926 and AB771927.

Corresponding author: Shigeyuki Kawano

Mailing address: Integrated Biosciences, Graduate School of Frontier Sciences,

University of Tokyo, Kashiwanoha 5-1-5, Kashiwa, Chiba 277-8562, Japan

Phone: +81-4-7136-3673

FAX: +81-4-7136-3674

Email: kawano@k.u-tokyo.ac.jp

DOI: 10.1534/g3.113.008599

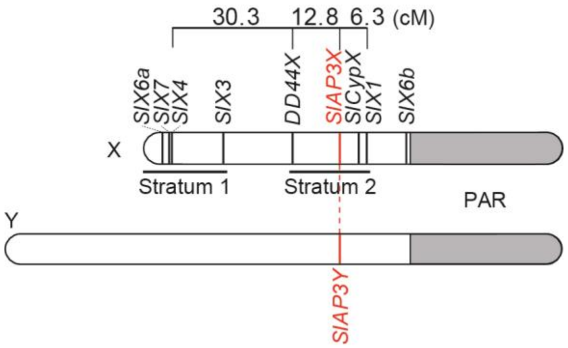

Figure S1 Genetic mapping of X-linked genes. The X-linkage map was constructed from the recombination values given in Table S7. The distances between each of the four X-linked genes (*SlX1*, *SlAP3X*, *DD44X*, and *SlX4*) correspond to those between the adjacent genes. The positions of the other six X-linked genes and the pseudoautosomal region (PAR) are based on the previously published map (Bergero *et al.* 2007).

Table S1 Summary of BAC sequencing

BAC	Raw sequence (reads)	Total number of bases (bp)	Number of contigs	Largest contig size (bp)	Number of bases (bp)
7a8D	84,298	31,817,620	412	20,096	249,797
13d11Ea	45,155	17,201,793	635	40,768	397,768
13d11Eb	41,083	17,256,042	1,347	24,376	808,699

Table S2 Primer sets used for Southern blot analysis of introns

Amplified region	Forward primer sequence (5'-3')	Reverse primer sequence (5'-3')
SlAP3X intron 1	GTACGTCTTTTTTTCATACCATCCCGTGG	AATTTGAATAACAATTAGCGTTAGGGTCCG
SlAP3X intron 2	TGGTGTCAAATGATCGAAGCGCAAAAC	CCAATATCAACATATTATTGAGCTTTAGC
SlAP3X intron 3	CTTTACAAAGACTATTAATTCCTACTAAC	CTATCATGATGCAAATTAATACAGAATC
SlAP3X intron 4	GTGAATTTCTTTTATGTACAAAATCATGG	CTGCAATTAACACGTTTTCAGAGCC
SlAP3X intron 5	GTAAGTAATCACCCCCCTACAAAAAAC	CTACATTTTCAAAACACCCACATTTACAAG
SlAP3Y intron 1	GTCTTTTTTTCTAAACACCCGATAATTCC	CTTAATGATCAAATAACAACGACAAAAAAG
SlAP3Y intron 3	GTGACCTTTTAAACAGGTTTTTTTTTTTTT	CTACAATGCAGCAAATTAAAATAGCATC
SlAP3Y intron 4	GTATAAAGTCATGCATTACTAAATCTCCTC	CATTTACTAATTTAAGTAGTTTCATACACG
SlAP3Y intron 5	GTTAGCTAATTTGTCACTTTTATAATTCAC	CTGAAAAGAGTGAAAAGGATTGATAATGG
SlAP3Y intron 6	GTAAGTAGTTTACTACTACGAAATCTTG	CTTCATTTTCAGAACTATTAGTTAGACTCG

Table S3 Primer sets used for X chromosome linkage mapping

Amplified gene	Forward primer sequence (5'-3')	Reverse primer sequence (5'-3')
SlAP3X	TCTGCTCTTGTGACTCTGTGTTTT	ACAATGGTGTTGCTCGACATGG
SlX1	GGTTTTGGCTACACCATTCGGGT	GTTCATTCGGGTCAAGTCAGTACA
SlX4	GTACACCCCGGAAATTTTGGGC	CGATACAAGTTGACCCGCTTGAGA
DD44X	CCCTGTCGTTCGAAATGCTCTT	CTCCGACTCAACCTTGTTCAATTCC

Underlined nucleotides are the polymorphic sites.

Table S4 Result of TBLASTX search using 13d11E as the query sequence

Accession Num.	Type	Start (bp)	End (bp)	Length (bp)	Frame	Homology	E-value	Identity (%)
D85597	Copia	3	38	36	-1	RH	0	43
		31	1221	1191	-3	RT	0	67
		1231	1290	60	-3	RT	0	50
		1390	2313	924	-3	PR/IN	0	64
		2338	2706	369	-3	PR	0	46
		2752	2820	69	-3	gag	0	43
EU646427.1	Gypsy	6456	6668	213	2	IN	5.00E-37	94
EU646427.1	Gypsy	7471	7650	180	-1	IN	3.00E-33	83
HE598754.1	Gypsy	15015	15116	102	-1	gag-pol	1.00E-49	44
		15336	15563	228	-1	gag-pol	1.00E-49	57
		15555	15713	159	-1	gag-pol	1.00E-49	62
		15705	15830	126	-1	gag-pol	1.00E-49	48
		16035	16124	90	-1	gag-pol	1.00E-49	40
XM_002276845.1	Gypsy	19019	19498	480	1	RH	0	50
		19505	19567	63	1	RH	0	62
		19566	19682	117	2	RH	0	42
		19691	20968	1278	1	RH/IN	0	57
		20975	20988	14	1	none	0	88

XM_002266431.1	Gypsy	20015	20968	954	3	IN	1.00E-158	62
		20975	21136	162	3	none	1.00E-158	60
EU646318.1	Copia	37024	37170	147	-3	none	2.00E-45	76
		37292	37378	87	-2	none	2.00E-45	78
		37407	37547	141	-1	RT	2.00E-45	85
XM_002266431.1	Gypsy	44378	44518	141	-1	none	0	56
		44509	45315	807	-2	IN	0	58
		45303	45590	288	-3	none	0	45
		45605	45958	354	-1	RH	0	49
		45965	46033	69	-1	RH	0	57
		46027	46065	39	-2	RH	0	62
		46120	46449	330	-2	RH	0	50
		46456	47580	1125	-2	RT/RH	0	64
XM_002274922.1	Gypsy	48460	48534	75	-3	none	6.00E-69	48
		48697	49206	510	-3	PR	6.00E-69	42
		49279	49392	114	-3	none	6.00E-69	58
		50056	50190	135	-3	none	6.00E-69	38
		50545	50844	300	-3	gag	6.00E-69	32
AB072493.1	Gypsy	55565	55915	351	1	gag	1.00E-43	60
AY172035.1	DNA	52707	52769	63	-2	transposase	1.00E-92	86
	transposon	52761	52823	63	-2	transposase	1.00E-92	77
		52846	52899	54	-1	transposase	1.00E-92	56

		52891	53085	195	-1	transposase	1.00E-92	80
		53079	53129	51	-2	transposase	1.00E-92	71
		53178	53294	117	-2	transposase	1.00E-92	75
		53339	53464	126	-3	transposase	1.00E-92	29
		53581	53649	69	-1	transposase	1.00E-92	70
		53656	53721	66	-1	transposase	1.00E-92	55
		53707	53772	66	-1	transposase	1.00E-92	60
		53773	53832	60	-1	transposase	1.00E-92	50
		53853	53942	90	-2	transposase	1.00E-92	67
		53927	54004	78	-3	transposase	1.00E-92	50
AB111100	Gypsy	56006	56203	198	3	gag	1.00E-155	23
		56441	56584	144	3	none	1.00E-155	34
		56825	57256	432	3	PR	1.00E-155	42
		57419	58345	927	3	RT	1.00E-155	57
		58406	58459	54	3	RH	1.00E-155	62
AB242301.1	Gypsy	64160	64459	300	1	RH	0	49
		64502	64966	465	1	RH/IN	0	41
		65117	65977	861	1	none	0	52
		66011	66328	318	1	none	0	47
XM_002274452.1	Copia	71622	71828	207	1	IN	5.00E-78	47
		71828	72031	204	3	none	5.00E-78	46
		72490	72996	507	2	RT	5.00E-78	47
EF101866.1	Copia	72897	73316	420	-1	RT	0	46

		73323	73385	63	-1	RT	0	71
		73541	73699	159	-2	RT	0	53
		73770	73871	102	-1	RT	0	59
		75035	75154	120	-2	IN	0	83
		75155	75451	297	-2	IN	0	75
		75452	75733	282	-2	IN	0	54
		75806	76534	729	-2	IN	0	60
		76891	76998	108	-3	IN	0	64
AY833550.1	DNA	82803	83225	423	-3	transposase	3.00E-84	46
	transposon	83244	83378	135	-3	transposase	3.00E-84	49
		83387	83608	222	-1	transposase	3.00E-84	49
		83639	83857	219	-1	transposase	3.00E-84	37
		83894	84061	168	-1	transposase	3.00E-84	30
AF039376.1	Copia	88463	88588	126	1	RH	0	48
		88661	88858	198	1	RH	0	38
		89214	89267	54	2	RT	0	44
		89403	89564	162	2	RT	0	54
		89583	89747	165	2	RT	0	40
		89799	89870	72	2	none	0	58
		89889	90644	756	2	IN	0	58
		90957	91025	69	2	none	0	44
		91032	92393	1362	2	none	0	54
EF101866.1	DNA	102068	102300	233	-1	none	0	50

	transposon	105435	105512	78	-3	none	0	69
		105525	105668	144	-3	none	0	46
		105666	105815	150	-3	none	0	48
		105813	105932	120	-3	none	0	35
		106039	106095	57	-2	none	0	47
		106271	106384	114	-1	none	0	47
		106403	106570	168	-1	none	0	66
		106556	106774	219	-1	none	0	79
		106764	106997	234	-3	none	0	67
		107076	107237	162	-3	none	0	65
		107331	107444	114	-3	none	0	58
		108019	108192	174	-2	transposase	0	64
		108183	108479	297	-3	transposase	0	73
		108486	108620	135	-3	transposase	0	51
		108624	108974	351	-3	transposase	0	49
EF101866.1	DNA transposon	110803	110994	192	1	RNA-directed DNA polymerase	4.00E-06	34
EF101866.1	DNA	112642	112749	108	-1	none	0	44
	transposon	112771	112866	96	-1	none	0	53
		112865	112942	78	-3	none	0	77
		112928	113368	441	-3	none	0	68
		113705	113821	117	-3	transposase	0	59
		113818	114288	471	-1	transposase	0	69

	114286	114420	135	-1	none	0	56
	114421	114771	351	-1	none	0	44

Table S5 Result of TBLASTX search using 7a8D as the query sequence

Accession Num.	Type	Start (bp)	End (bp)	Length (bp)	Frame	Homology	E-value	Identity (%)
HE598754.1	Gypsy	377	442	66	-2	gag-pol	3.00E-57	55
		701	742	42	-2	gag-pol	3.00E-57	72
		758	859	102	-2	gag-pol	3.00E-57	42
		1079	1459	381	-2	gag-pol	3.00E-57	61
		1451	1576	126	-2	gag-pol	3.00E-57	48
AK229858.1	LINE	19096	19347	252	-1	RT	3.00E-15	42
FM993987.1	LINE	32034	32291	258	1	RT/RH	9.00E-39	56
		32797	33069	273	2	RT/RH	9.00E-39	41
XM_002264986.1	Gypsy	42017	43255	1239	-3	IN	0.00E+00	55
		43328	44500	1173	-3	RT/RH	0.00E+00	65
HE598766.1	Gypsy	49861	50187	327	-3	env	6.00E-15	43
FJ197984.1	Gypsy	52892	52996	105	-3	gag-pol	7.00E-100	37
		54288	54374	87	-2	gag-pol	7.00E-100	66
		54387	54671	285	-2	gag-pol	7.00E-100	51
		54739	54882	144	-1	gag-pol	7.00E-100	58
		55102	55263	162	-1	gag-pol	7.00E-100	35
		55909	56445	537	-1	gag-pol	7.00E-100	45
		56458	56529	72	-1	gag-pol	7.00E-100	46
AB111100.1	Gypsy	63023	63142	120	2	none	3.00E-88	40

		63173	63391	219	2	gag	3.00E-88	34
		63405	63563	159	3	gag	3.00E-88	19
		63800	63946	147	2	none	3.00E-88	35
		64151	64546	396	2	PR	3.00E-88	41
AB242301.1	Gypsy	64624	65379	756	3	RT	0.00E+00	56
		65395	65688	294	3	RH	0.00E+00	48
		65755	65877	123	3	RH	0.00E+00	56
		65866	66132	267	3	RH	0.00E+00	44
		67569	67946	378	2	none	0.00E+00	41
		67963	68142	180	3	IN	0.00E+00	50
		68515	68829	315	3	none	0.00E+00	50

Table S6 Result of BLASTN searches

Query	Name	Start (bp)	End (bp)	Length (bp)	Frame	Score (bit)	E-value	Identity (%)
13d11E	Copia-13_SB-I Copia Sorghum bicolor	1	2258	2258	-	742	0	68
(*SlAP3X*)	Copia25-PTR_I Copia Populus trichocarpa	1360	2270	911	-	356	8.00E-96	70
	Ogre-MT4_I Gypsy Medicago truncatula	20134	20759	626	+	91	5.00E-16	65
	Ogre-VP1_I Gypsy Vicia pannonica	20251	20729	479	+	154	5.00E-35	68
	Gyp_I_MT Gypsy Medicago truncatula	20251	20769	519	+	102	3.00E-19	66
	Ogre-VP1_I Gypsy Vicia pannonica	45013	45242	230	-	87	6.00E-15	70
	Ogre-MT4_I Gypsy Medicago truncatula	45032	45308	277	-	80	9.00E-13	68
	Ogre-LE1_I Gypsy Solanum lycopersicum	46524	47354	831	-	98	3.00E-18	63
	Ogre-VP1_I Gypsy Vicia pannonica	46728	47516	789	-	197	5.00E-48	66
	Ogre-MT3_I Gypsy Medicago truncatula	46914	47518	605	-	179	1.00E-42	67
	ATLANTYS1_I Gypsy Arabidopsis thaliana	57606	58358	753	+	206	9.00E-51	67
	Gypsy3-VV_I Gypsy Vitis vinifera	65228	65517	290	+	75	4.00E-11	67
	Copia35-PTR_I Copia Populus trichocarpa	73582	73876	295	+	105	2.00E-20	69
	Copia-77_SB-I Copia Sorghum bicolor	74906	76232	1327	-	295	2.00E-77	66
	COP3_I_MT Copia Medicago truncatula	74910	76460	1551	-	645	0	70
	COP_I_MT Copia Medicago truncatula	76824	78172	1349	-	224	3.00E-56	65
	COP3_I_MT Copia Medicago truncatula	76889	78125	1237	-	284	4.00E-74	67
	Copia40-PTR_I Copia Populus trichocarpa	89909	90549	641	+	129	2.00E-27	66
	Copia8-PTR_I Copia Populus trichocarpa	90208	90623	416	+	87	6.00E-15	66

	Copia35-ZM_I	Copia	Zea mays	91147	91506	360	+	107	7.00E-21	67
	ATCOPIA4I	Copia	Arabidopsis thaliana	91203	91638	436	+	127	7.00E-27	67
	ATCOPIA5I	Copia	Arabidopsis thaliana	92090	92503	414	+	80	9.00E-13	66
	ATENSPM9	EnSpm	Arabidopsis thaliana	106561	106696	136	-	104	8.00E-20	79
	EnSpm3_PT	EnSpm	Populus trichocarpa	106562	106873	312	-	120	1.00E-24	70
	EnSpm2_PTr	EnSpm	Populus trichocarpa	108060	108469	410	-	138	4.00E-30	68
	ATENSPM9	EnSpm	Arabidopsis thaliana	112933	113079	147	-	86	2.00E-14	73
	EnSpm3_PT	EnSpm	Populus trichocarpa	112934	113244	311	-	96	1.00E-17	68
	ENSPM1_PT	EnSpm	Populus trichocarpa	113816	114258	443	-	134	5.00E-29	67
	ENSPM_AC	EnSpm	Allium cepa	114090	114269	180	-	86	2.00E-14	72
7a8D (*SlAP3Y*)	Gypsy-44_BD-I	Gypsy	Brachypodium distachyon	42224	42921	698	-	113	1.00E-22	65
	Gypsy18-PTR_I	Gypsy	Populus trichocarpa	42245	44394	2150	-	426	3.00E-117	66
	Gypsy-16_Mad-I	Gypsy	Malus x domestica	42245	44621	2377	-	343	3.00E-92	65
	Gypsy7-VV_I	Gypsy	Vitis vinifera	43352	44660	1309	-	219	9.00E-55	65
	POPGY1_I	Gypsy	Populus trichocarpa	43541	44826	1286	-	161	2.00E-37	64
	Gypsy3-VV_I	Gypsy	Vitis vinifera	64561	64828	268	+	75	2.00E-11	68
	ATLANTYS2_I	Gypsy	Arabidopsis thaliana	64576	65335	760	+	150	4.00E-34	66
	ATLANTYS1_I	Gypsy	Arabidopsis thaliana	64576	65419	844	+	141	2.00E-31	65

Table S7 Number and proportion (in parentheses) of recombinants (above the diagonal) and genetic distance (in cM, below the diagonal) between the four X-linked genes

	SlX1	*SlAP3X*	*DD44X*	*SlX4*
SlX1	-	6 (0.06)**	16 (0.17)**	34 (0.35)*
SlAP3X	6.3	-	12 (0.13)**	32 (0.33)**
DD44X	17.3	12.8	-	26 (0.27)**
SlX4	44.2	40.2	30.3	-

Asterisks indicate the significance of linkage (deviation from independent segregation) as determined by the G-test. *P < 0.01; **P < 0.001
