# Supplementary material for: Rapid Degeneration of Noncoding DNA Regions Surrounding SlAP3X/Y After Recombination Suppression in the Dioecious Plant Silene latifolia
Source: G3 (Bethesda). 2013 Oct 11;3(12):2121–30. doi: 10.1534/g3.113.008599 (PMC3852375; doi:10.1534/g3.113.008599)
Supplement: Supporting Information [file supp_g3.113.008599_FigureS1.pdf]

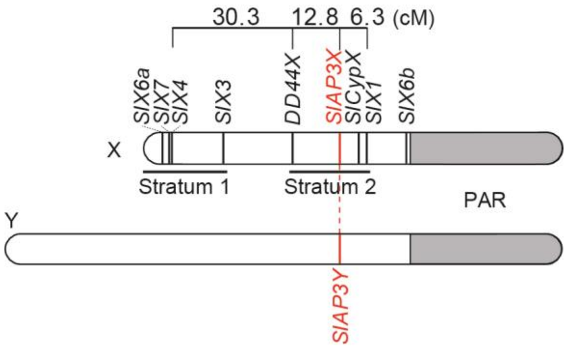

Figure S1 Genetic mapping of X-linked genes. The X-linkage map was constructed from the recombination values given in Table S7. The distances between each of the four X-linked genes (*SlX1*, *SlAP3X*, *DD44X*, and *SlX4*) correspond to those between the adjacent genes. The positions of the other six X-linked genes and the pseudoautosomal region (PAR) are based on the previously published map (Bergero *et al.* 2007).
