# Supplementary material for: Rapid Degeneration of Noncoding DNA Regions Surrounding SlAP3X/Y After Recombination Suppression in the Dioecious Plant Silene latifolia
Source: G3 (Bethesda). 2013 Oct 11;3(12):2121–30. doi: 10.1534/g3.113.008599 (PMC3852375; doi:10.1534/g3.113.008599)
Supplement: Supporting Information [file supp_g3.113.008599_TableS1.pdf]

Table S1 Summary of BAC sequencing

BAC	Raw sequence (reads)	Total number of bases (bp)	Number of contigs	Largest contig size (bp)	Number of bases (bp)
7a8D	84,298	31,817,620	412	20,096	249,797
13d11Ea	45,155	17,201,793	635	40,768	397,768
13d11Eb	41,083	17,256,042	1,347	24,376	808,699
