# Supplementary material for: Rapid Degeneration of Noncoding DNA Regions Surrounding SlAP3X/Y After Recombination Suppression in the Dioecious Plant Silene latifolia
Source: G3 (Bethesda). 2013 Oct 11;3(12):2121–30. doi: 10.1534/g3.113.008599 (PMC3852375; doi:10.1534/g3.113.008599)
Supplement: Supporting Information [file supp_g3.113.008599_TableS2.pdf]

**Table S2 Primer sets used for Southern blot analysis of introns**

| Amplified region | Forward primer sequence (5'-3') | Reverse primer sequence (5'-3') |
|---|---|---|
| *SlAP3X* intron 1 | GTACGTCTTTTTTTCATACCATCCCGTGG | AATTTGAATAACAATTAGCGTTAGGGTCCG |
| *SlAP3X* intron 2 | TGGTGTCAAATGATCGAAGCGCAAAAC | CCAATATCAACATATTATTGAGCTTTAGC |
| *SlAP3X* intron 3 | CTTTACAAAGACTATTAATTCCTACTAAC | CTATCATGATGCAAATTAATACAGAATC |
| *SlAP3X* intron 4 | GTGAATTTCTTTTATGTACAAAATCATGG | CTGCAATTAACACGTTTTCAGAGCC |
| *SlAP3X* intron 5 | GTAAGTAATCACCCCCCTACAAAAAAC | CTACATTTTCAAAACACCCACATTTACAAG |
| *SlAP3Y* intron 1 | GTCTTTTTTTCTAAACACCCGATAATTCC | CTTAATGATCAAATAACAACGACAAAAAAG |
| *SlAP3Y* intron 3 | GTGACCTTTTAAACAGGTTTTTTTTTTTTT | CTACAATGCAGCAAATTAAAATAGCATC |
| *SlAP3Y* intron 4 | GTATAAAGTCATGCATTACTAAATCTCCTC | CATTTACTAATTTAAGTAGTTTCATACACG |
| *SlAP3Y* intron 5 | GTTAGCTAATTTGTCACTTTTATAATTCAC | CTGAAAAGAGTGAAAAGGATTGATAATGG |
| *SlAP3Y* intron 6 | GTAAGTAGTTTACTACTACGAAATCTTG | CTTCATTTTCAGAACTATTAGTTAGACTCG |
