# Supplementary material for: Rapid Degeneration of Noncoding DNA Regions Surrounding SlAP3X/Y After Recombination Suppression in the Dioecious Plant Silene latifolia
Source: G3 (Bethesda). 2013 Oct 11;3(12):2121–30. doi: 10.1534/g3.113.008599 (PMC3852375; doi:10.1534/g3.113.008599)
Supplement: Supporting Information [file supp_g3.113.008599_TableS3.pdf]

Table S3 Primer sets used for X chromosome linkage mapping

Amplified gene	Forward primer sequence (5'-3')	Reverse primer sequence (5'-3')
SlAP3X	TCTGCTCTTGTGACTCTGTGTTTT	ACAATGGTGTTGCTCGACATGG
SlX1	GGTTTTGGCTACACCATTCGGGT	GTTCATTCGGGTCAAGTCAGTACA
SlX4	GTACACCCCGGAAATTTTGGGC	CGATACAAGTTGACCCGCTTGAGA
DD44X	CCCTGTCGTTCGAAATGCTCTT	CTCCGACTCAACCTTGTTCAATTCC

Underlined nucleotides are the polymorphic sites.
