# Supplementary material for: Rapid Degeneration of Noncoding DNA Regions Surrounding SlAP3X/Y After Recombination Suppression in the Dioecious Plant Silene latifolia
Source: G3 (Bethesda). 2013 Oct 11;3(12):2121–30. doi: 10.1534/g3.113.008599 (PMC3852375; doi:10.1534/g3.113.008599)
Supplement: Supporting Information [file supp_g3.113.008599_TableS4.pdf]

Table S4 Result of TBLASTX search using 13d11E as the query sequence

Accession Num.	Type	Start (bp)	End (bp)	Length (bp)	Frame	Homology	E-value	Identity (%)
D85597	Copia	3	38	36	-1	RH	0	43
		31	1221	1191	-3	RT	0	67
		1231	1290	60	-3	RT	0	50
		1390	2313	924	-3	PR/IN	0	64
		2338	2706	369	-3	PR	0	46
		2752	2820	69	-3	gag	0	43
EU646427.1	Gypsy	6456	6668	213	2	IN	5.00E-37	94
EU646427.1	Gypsy	7471	7650	180	-1	IN	3.00E-33	83
HE598754.1	Gypsy	15015	15116	102	-1	gag-pol	1.00E-49	44
		15336	15563	228	-1	gag-pol	1.00E-49	57
		15555	15713	159	-1	gag-pol	1.00E-49	62
		15705	15830	126	-1	gag-pol	1.00E-49	48
		16035	16124	90	-1	gag-pol	1.00E-49	40
XM_002276845.1	Gypsy	19019	19498	480	1	RH	0	50
		19505	19567	63	1	RH	0	62
		19566	19682	117	2	RH	0	42
		19691	20968	1278	1	RH/IN	0	57
		20975	20988	14	1	none	0	88

XM_002266431.1	Gypsy	20015	20968	954	3	IN	1.00E-158	62
		20975	21136	162	3	none	1.00E-158	60
EU646318.1	Copia	37024	37170	147	-3	none	2.00E-45	76
		37292	37378	87	-2	none	2.00E-45	78
		37407	37547	141	-1	RT	2.00E-45	85
XM_002266431.1	Gypsy	44378	44518	141	-1	none	0	56
		44509	45315	807	-2	IN	0	58
		45303	45590	288	-3	none	0	45
		45605	45958	354	-1	RH	0	49
		45965	46033	69	-1	RH	0	57
		46027	46065	39	-2	RH	0	62
		46120	46449	330	-2	RH	0	50
		46456	47580	1125	-2	RT/RH	0	64
XM_002274922.1	Gypsy	48460	48534	75	-3	none	6.00E-69	48
		48697	49206	510	-3	PR	6.00E-69	42
		49279	49392	114	-3	none	6.00E-69	58
		50056	50190	135	-3	none	6.00E-69	38
		50545	50844	300	-3	gag	6.00E-69	32
AB072493.1	Gypsy	55565	55915	351	1	gag	1.00E-43	60
AY172035.1	DNA transposon	52707	52769	63	-2	transposase	1.00E-92	86
		52761	52823	63	-2	transposase	1.00E-92	77
		52846	52899	54	-1	transposase	1.00E-92	56

		52891	53085	195	-1	transposase	1.00E-92	80
		53079	53129	51	-2	transposase	1.00E-92	71
		53178	53294	117	-2	transposase	1.00E-92	75
		53339	53464	126	-3	transposase	1.00E-92	29
		53581	53649	69	-1	transposase	1.00E-92	70
		53656	53721	66	-1	transposase	1.00E-92	55
		53707	53772	66	-1	transposase	1.00E-92	60
		53773	53832	60	-1	transposase	1.00E-92	50
		53853	53942	90	-2	transposase	1.00E-92	67
		53927	54004	78	-3	transposase	1.00E-92	50
AB111100	Gypsy	56006	56203	198	3	gag	1.00E-155	23
		56441	56584	144	3	none	1.00E-155	34
		56825	57256	432	3	PR	1.00E-155	42
		57419	58345	927	3	RT	1.00E-155	57
		58406	58459	54	3	RH	1.00E-155	62
AB242301.1	Gypsy	64160	64459	300	1	RH	0	49
		64502	64966	465	1	RH/IN	0	41
		65117	65977	861	1	none	0	52
		66011	66328	318	1	none	0	47
XM_002274452.1	Copia	71622	71828	207	1	IN	5.00E-78	47
		71828	72031	204	3	none	5.00E-78	46
		72490	72996	507	2	RT	5.00E-78	47
EF101866.1	Copia	72897	73316	420	-1	RT	0	46

		73323	73385	63	-1	RT	0	71
		73541	73699	159	-2	RT	0	53
		73770	73871	102	-1	RT	0	59
		75035	75154	120	-2	IN	0	83
		75155	75451	297	-2	IN	0	75
		75452	75733	282	-2	IN	0	54
		75806	76534	729	-2	IN	0	60
		76891	76998	108	-3	IN	0	64
AY833550.1	DNA	82803	83225	423	-3	transposase	3.00E-84	46
	transposon	83244	83378	135	-3	transposase	3.00E-84	49
		83387	83608	222	-1	transposase	3.00E-84	49
		83639	83857	219	-1	transposase	3.00E-84	37
		83894	84061	168	-1	transposase	3.00E-84	30
AF039376.1	Copia	88463	88588	126	1	RH	0	48
		88661	88858	198	1	RH	0	38
		89214	89267	54	2	RT	0	44
		89403	89564	162	2	RT	0	54
		89583	89747	165	2	RT	0	40
		89799	89870	72	2	none	0	58
		89889	90644	756	2	IN	0	58
		90957	91025	69	2	none	0	44
		91032	92393	1362	2	none	0	54
EF101866.1	DNA	102068	102300	233	-1	none	0	50

	transposon	105435	105512	78	-3	none	0	69
		105525	105668	144	-3	none	0	46
		105666	105815	150	-3	none	0	48
		105813	105932	120	-3	none	0	35
		106039	106095	57	-2	none	0	47
		106271	106384	114	-1	none	0	47
		106403	106570	168	-1	none	0	66
		106556	106774	219	-1	none	0	79
		106764	106997	234	-3	none	0	67
		107076	107237	162	-3	none	0	65
		107331	107444	114	-3	none	0	58
		108019	108192	174	-2	transposase	0	64
		108183	108479	297	-3	transposase	0	73
		108486	108620	135	-3	transposase	0	51
		108624	108974	351	-3	transposase	0	49
EF101866.1	DNA transposon	110803	110994	192	1	RNA-directed DNA polymerase	4.00E-06	34
EF101866.1	DNA transposon	112642	112749	108	-1	none	0	44
		112771	112866	96	-1	none	0	53
		112865	112942	78	-3	none	0	77
		112928	113368	441	-3	none	0	68
		113705	113821	117	-3	transposase	0	59
		113818	114288	471	-1	transposase	0	69

	114286	114420	135	-1	none	0	56
	114421	114771	351	-1	none	0	44
