# Supplementary material for: Rapid Degeneration of Noncoding DNA Regions Surrounding SlAP3X/Y After Recombination Suppression in the Dioecious Plant Silene latifolia
Source: G3 (Bethesda). 2013 Oct 11;3(12):2121–30. doi: 10.1534/g3.113.008599 (PMC3852375; doi:10.1534/g3.113.008599)
Supplement: Supporting Information [file supp_g3.113.008599_TableS5.pdf]

Table S5 Result of TBLASTX search using 7a8D as the query sequence

Accession Num.	Type	Start (bp)	End (bp)	Length (bp)	Frame	Homology	E-value	Identity (%)
HE598754.1	Gypsy	377	442	66	-2	gag-pol	3.00E-57	55
		701	742	42	-2	gag-pol	3.00E-57	72
		758	859	102	-2	gag-pol	3.00E-57	42
		1079	1459	381	-2	gag-pol	3.00E-57	61
		1451	1576	126	-2	gag-pol	3.00E-57	48
AK229858.1	LINE	19096	19347	252	-1	RT	3.00E-15	42
FM993987.1	LINE	32034	32291	258	1	RT/RH	9.00E-39	56
		32797	33069	273	2	RT/RH	9.00E-39	41
XM_002264986.1	Gypsy	42017	43255	1239	-3	IN	0.00E+00	55
		43328	44500	1173	-3	RT/RH	0.00E+00	65
HE598766.1	Gypsy	49861	50187	327	-3	env	6.00E-15	43
FJ197984.1	Gypsy	52892	52996	105	-3	gag-pol	7.00E-100	37
		54288	54374	87	-2	gag-pol	7.00E-100	66
		54387	54671	285	-2	gag-pol	7.00E-100	51
		54739	54882	144	-1	gag-pol	7.00E-100	58
		55102	55263	162	-1	gag-pol	7.00E-100	35
		55909	56445	537	-1	gag-pol	7.00E-100	45
		56458	56529	72	-1	gag-pol	7.00E-100	46
AB111100.1	Gypsy	63023	63142	120	2	none	3.00E-88	40

		63173	63391	219	2	gag	3.00E-88	34
		63405	63563	159	3	gag	3.00E-88	19
		63800	63946	147	2	none	3.00E-88	35
		64151	64546	396	2	PR	3.00E-88	41
AB242301.1	Gypsy	64624	65379	756	3	RT	0.00E+00	56
		65395	65688	294	3	RH	0.00E+00	48
		65755	65877	123	3	RH	0.00E+00	56
		65866	66132	267	3	RH	0.00E+00	44
		67569	67946	378	2	none	0.00E+00	41
		67963	68142	180	3	IN	0.00E+00	50
		68515	68829	315	3	none	0.00E+00	50
