# Supplementary material for: Rapid Degeneration of Noncoding DNA Regions Surrounding SlAP3X/Y After Recombination Suppression in the Dioecious Plant Silene latifolia
Source: G3 (Bethesda). 2013 Oct 11;3(12):2121–30. doi: 10.1534/g3.113.008599 (PMC3852375; doi:10.1534/g3.113.008599)
Supplement: Supporting Information [file supp_g3.113.008599_TableS6.pdf]

Table S6 Result of BLASTN searches

Query	Name	Start (bp)	End (bp)	Length (bp)	Frame	Score (bit)	E-value	Identity (%)
13d11E (*SlAP3X*)	Copia-13_SB-I Copia Sorghum bicolor	1	2258	2258	-	742	0	68
	Copia25-PTR_I Copia Populus trichocarpa	1360	2270	911	-	356	8.00E-96	70
	Ogre-MT4_I Gypsy Medicago truncatula	20134	20759	626	+	91	5.00E-16	65
	Ogre-VP1_I Gypsy Vicia pannonica	20251	20729	479	+	154	5.00E-35	68
	Gyp_I_MT Gypsy Medicago truncatula	20251	20769	519	+	102	3.00E-19	66
	Ogre-VP1_I Gypsy Vicia pannonica	45013	45242	230	-	87	6.00E-15	70
	Ogre-MT4_I Gypsy Medicago truncatula	45032	45308	277	-	80	9.00E-13	68
	Ogre-LE1_I Gypsy Solanum lycopersicum	46524	47354	831	-	98	3.00E-18	63
	Ogre-VP1_I Gypsy Vicia pannonica	46728	47516	789	-	197	5.00E-48	66
	Ogre-MT3_I Gypsy Medicago truncatula	46914	47518	605	-	179	1.00E-42	67
	ATLANTYS1_I Gypsy Arabidopsis thaliana	57606	58358	753	+	206	9.00E-51	67
	Gypsy3-VV_I Gypsy Vitis vinifera	65228	65517	290	+	75	4.00E-11	67
	Copia35-PTR_I Copia Populus trichocarpa	73582	73876	295	+	105	2.00E-20	69
	Copia-77_SB-I Copia Sorghum bicolor	74906	76232	1327	-	295	2.00E-77	66
	COP3_I_MT Copia Medicago truncatula	74910	76460	1551	-	645	0	70
	COP_I_MT Copia Medicago truncatula	76824	78172	1349	-	224	3.00E-56	65
	COP3_I_MT Copia Medicago truncatula	76889	78125	1237	-	284	4.00E-74	67
	Copia40-PTR_I Copia Populus trichocarpa	89909	90549	641	+	129	2.00E-27	66
	Copia8-PTR_I Copia Populus trichocarpa	90208	90623	416	+	87	6.00E-15	66

	Copia35-ZM_I Copia Zea mays	91147	91506	360	+	107	7.00E-21	67
	ATCOPIA4I Copia Arabidopsis thaliana	91203	91638	436	+	127	7.00E-27	67
	ATCOPIA5I Copia Arabidopsis thaliana	92090	92503	414	+	80	9.00E-13	66
	ATENSPM9 EnSpm Arabidopsis thaliana	106561	106696	136	-	104	8.00E-20	79
	EnSpm3_PT EnSpm Populus trichocarpa	106562	106873	312	-	120	1.00E-24	70
	EnSpm2_PTr EnSpm Populus trichocarpa	108060	108469	410	-	138	4.00E-30	68
	ATENSPM9 EnSpm Arabidopsis thaliana	112933	113079	147	-	86	2.00E-14	73
	EnSpm3_PT EnSpm Populus trichocarpa	112934	113244	311	-	96	1.00E-17	68
	ENSPM1_PT EnSpm Populus trichocarpa	113816	114258	443	-	134	5.00E-29	67
	ENSPM_AC EnSpm Allium cepa	114090	114269	180	-	86	2.00E-14	72
7a8D (*SlAP3Y*)	Gypsy-44_BD-I Gypsy Brachypodium distachyon	42224	42921	698	-	113	1.00E-22	65
	Gypsy18-PTR_I Gypsy Populus trichocarpa	42245	44394	2150	-	426	3.00E-117	66
	Gypsy-16_Mad-I Gypsy Malus x domestica	42245	44621	2377	-	343	3.00E-92	65
	Gypsy7-VV_I Gypsy Vitis vinifera	43352	44660	1309	-	219	9.00E-55	65
	POPGY1_I Gypsy Populus trichocarpa	43541	44826	1286	-	161	2.00E-37	64
	Gypsy3-VV_I Gypsy Vitis vinifera	64561	64828	268	+	75	2.00E-11	68
	ATLANTYS2_I Gypsy Arabidopsis thaliana	64576	65335	760	+	150	4.00E-34	66
	ATLANTYS1_I Gypsy Arabidopsis thaliana	64576	65419	844	+	141	2.00E-31	65
