# Supplementary material for: Rapid Degeneration of Noncoding DNA Regions Surrounding SlAP3X/Y After Recombination Suppression in the Dioecious Plant Silene latifolia
Source: G3 (Bethesda). 2013 Oct 11;3(12):2121–30. doi: 10.1534/g3.113.008599 (PMC3852375; doi:10.1534/g3.113.008599)
Supplement: Supporting Information [file supp_g3.113.008599_TableS7.pdf]

Table S7 Number and proportion (in parentheses) of recombinants (above the diagonal) and genetic distance (in cM, below the diagonal) between the four X-linked genes

	SlX1	*SlAP3X*	*DD44X*	*SlX4*
SlX1	-	6 (0.06)**	16 (0.17)**	34 (0.35)*
SlAP3X	6.3	-	12 (0.13)**	32 (0.33)**
DD44X	17.3	12.8	-	26 (0.27)**
SlX4	44.2	40.2	30.3	-

Asterisks indicate the significance of linkage (deviation from independent segregation) as determined by the G-test. *$P < 0.01$; **$P < 0.001$
